# A Meta‐Analysis of the Efficacy and Safety of Botulinum Toxin Type A for the Management of Scars After Facial Surgery

**DOI:** 10.1111/jocd.70111

**Published:** 2025-03-17

**Authors:** Aibo Jiang, Ruiming Jiang, Ting Liu

**Affiliations:** ^1^ Department of Dermatology Affiliated Hospital of North Sichuan Medical College Nanchong Sichuan P.R. China

**Keywords:** botulinum toxin A, facial cicatrix, facial scar, postoperative

## Abstract

**Background:**

Postoperative scarring of the face can cause a serious psychological burden on people. Botulinum toxin type A has shown potential effectiveness in preventing scarring after facial surgery. The study aims to evaluate the role of botulinum toxin type A in postoperative scar management.

**Methods:**

We searched PubMed, Embase, MEDLINE, the Cochrane Library (CENTRAL), and Web of Science for all randomized controlled trials (RCTs) on the use of botulinum toxin type A in the treatment of postoperative facial scars, including all English articles published up to April 15, 2024.

**Results:**

Twelve randomized controlled trials involving 351 patients undergoing facial surgery were included. Quantitative analysis, STATA 17.0 software was used for meta‐analysis, and fixed‐effect and random‐effects models were selected according to the size of heterogeneity, and the results were expressed as SMD and 95% CI for continuous data and OR and 95% CI for dichotomous data. Subgroup analyses were performed according to the different control groups. The OR and 95% CI of adverse effects were 1.74 (0.41–7.43). The *p* value of adverse reactions and PSAS results was greater than 0.05.

**Conclusion:**

Due to the small heterogeneity of scar width, OSAS, and PSAS, SMD and 95% CI were −1.00 (−1.20 to 0.80), −0.61 (−1.0 to 0.13), and −0.08 (−0.56 to 0.39), respectively, using a fixed‐effect model. Due to large heterogeneity, VAS and VSS scores were scored using a random‐effects model, with SMDs and 95% CIs of 1.00 (0.47–1.53) and −0.41 (−0.73 to 0.1), respectively.

**Trial Registration:**

PROSPERO number: CRD42024538070

## 1 Introduction

Whether it is trauma or postsurgical skin lesions for birth defects, these skin lesions inevitably lead to scarring. Facial scarring can have a significant negative impact on the patient's mental health because facial scars are different from body scars, which are covered by clothing, and facial scars cannot be completely hidden, so the main thing to reduce the burden of facial scars on patients is to reduce the facial scars and make them less conspicuous [1]. The tension at the edge of the wound plays a hindering role in the wound healing process, so tension is one of the significant factors affecting wound healing; thus, reducing the skin surface tension is an important factor affecting the final beauty effect of the scar [2]. The elastic retraction of the dermis and the atrophy of the musculature are the sources of skin tension [3]. The facial muscles are more tense, and the healing skin wound is constantly tense, leading to healing complications [4].

Many of the surgeries, lasers, radiation, and compression therapies used to treat facial scars are advanced, but none of them are very effective [5, 6, 7, 8]. Because the drug injection is less invasive to the skin and does not require high technology, it is widely used in the treatment of scars [9]. The case report also confirmed the healing effect of chemotherapy fixation on facial skin wounds [4]. In recent years, there have been many randomized controlled trials (RCT) of facial scars, but in general, the sample size of each study was relatively small [10, 11, 12, 13, 14, 15, 16, 17, 18, 19, 20]. Some studies have shown that botulinum toxin can significantly improve the shape and texture of scars and reduce the incidence of scarring, while others have not been conclusive. Therefore, there is a need for a systematic review and comprehensive analysis of existing studies to evaluate the role of botulinum toxin more comprehensively and objectively in postoperative scar management.

The purpose of this article is to comprehensively and systematically evaluate the efficacy and safety of botulinum toxin in the treatment of postoperative facial scars through meta‐analysis and to improve the evidence support for the clinical treatment and prevention of facial scars. We hope that through the in‐depth discussion of this study, we can provide patients with more effective postoperative scar management strategies and improve their quality of life and well‐being.

## 2 Methods

### 2.1 Search Strategy

We performed our meta‐analyses according to the Preferred Reporting Items for Systematic Reviews and Meta‐Analyses (PRISMA) standards checklist [21]. PubMed, Embase, MEDLINE, and the Cochrane Library (CENTRAL) were searched in the computer for published studies up to April 2024. English search terms include: (“botulinum” and “Botulinum Toxins, Type A” and “randomized controlled trials” and “randomized” and “RCT” and “face” and “cheek” and “eye” and “forehead” and “mouth” and “nose” and “clip” and “scar” and “cicatrix” and “facial scar” and “facial cicatrix”). Strictly follow the Mesh subject heading. Randomized controlled studies were used, with or without blinding and allocation concealment. To identify studies to be included, the above: The search strategy was constructed according to the PICOS principle: P (patients undergoing facial surgery), I (inject with BTX‐A), C (placebo‐treat or no treatment), O (improvement of postoperative scarring of the face). Here are the exclusion criteria for this article: (1) non‐English articles; (2) animal experiments; and (3) systematic reviews, case reports, reviews, editorials, conferences, letters, or opinions. See the Supporting Information for details.

### 2.2 Data Extraction

Two review staff independently screened the literature according to the inclusion and exclusion criteria. The extracted data included: (1) general characteristics: authors and time; (2) study characteristics: number of included patients, age, gender, loss to follow‐up, BTX‐A injection dose, treatment, and control measures; (3) outcome measures: observation time, outcome indicators, adverse reactions, etc.

### 2.3 Quality Assessment

Assessed according to the risk assessment methodology provided in the Cochrane manual, STATA 17 software pooled and generated a risk bias map. When there is a disagreement, the third researcher joins to discuss the decision.

### 2.4 Statistical Analysis

Data were meta‐analyzed using STATA 17. The *Q* statistic and the *I* ^2^ statistic were used for heterogeneity testing, and when *I* ^2^ < 50% and *p* > 0.1, a fixed‐effect model was used; otherwise, a random‐effects model would be used. Publication bias in meta‐analyses was assessed using funnel plots and Begg's test, and if *p* > 0.05, there was no publication bias; otherwise, there was publication bias. Standardized mean differences (SMDs) were used for continuous data and odds ratios (ORs) for dichotomous data, and a 95% confidence interval (95% CI) was calculated. The results were statistically significant with *p* < 0.05 as the difference.

## 3 Evidence Synthesis

### 3.1 Baseline Characteristics

**FIGURE 1:**
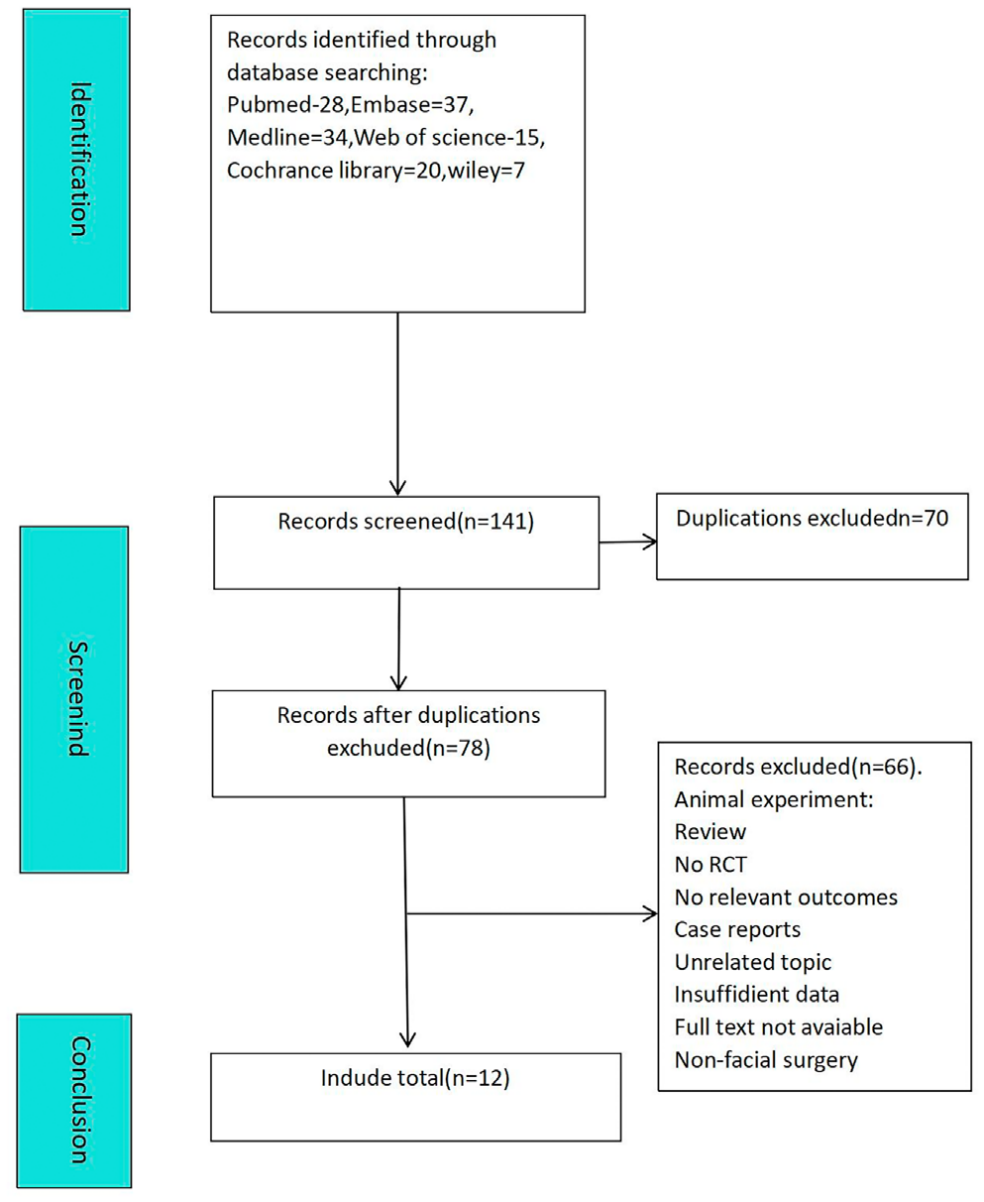
The process of selecting articles eligible for meta‐analysis. Ten of the studies compared BTA with normal saline, and two studies compared BTA with no treatment. The concentration of botulinum toxin type A in the experimental group ranged from 10 to 75 U/mL. Three of the articles were about birth defects, five were caused by surgery, and three were wounds caused by trauma.

A total of 141 articles were obtained from the literature search. In the end, 12 articles were included [1, 10, 11, 12, 13, 14, 15, 16, 17, 18, 19, 20]. The flowchart of literature screening is shown in Figure 1. Ten of the studies compared BTA with normal saline, and two studies compared BTA with no treatment. The concentration of botulinum toxin type A in the experimental group ranged from 10 to 75 U/mL. Three of the articles were about birth defects, five were caused by surgery, and three were wounds caused by trauma. The basic characteristics of the included studies are shown in Table 1.

**TABLE 1:** Basic characteristics of the included literature.

Author	Year	Design	Total		Loss rate	Age (mean ± SD)		Sex (M/F)		Concentration	Injection site	Injection time	Control	Wound	The type of wound	Follow‐up	Outcome indicators
			Experience	Control		Experience	Control	Experience	Control								
Sonane, J.	2022	RCT	12	10	21%	6.91 ± 0.65	7 ± 0.38	6/6	6/4	1	Adjacent orbicularis oris muscle	Immediately after completion of cleft lip repair	0.9% saline	Upper lip	Birth defects	6	VAS, VSS, scar width
Hu, L.	2018	RCT	7	7	12.50%	12.29 ± 11.64	12.29 ± 11.64	7/7	7/7	33.7	5 mm on either side of the wound	Immediately after wound closure	0.9% saline	Face	Surgery	6	VAS, VSS, scar width
Huang, Y.	2021	RCT	18	19	7.50%	61.56 ± 8.14	61.56 ± 8.14	2/16	4/15	7.5	Lateral orbicularis oculi muscles	Immediately after completion of surgery	0.9% saline	Lower eyelids	Surgery	7.10 ± 8.03:6.59 ± 8.36	VAS, VSS, scar width
Lin, M.	2022	RCT	20	20	0	58.08 ± 21.54	59.00 ± 25.53	12/8	11/9	50	Forehead	Immediately following the closure of Mohs defects	0.9% saline	Forehead	Surgery	1 week, 3 weeks, 6 month	MMSS, VAS, scar width
Huang, R.	2019	RCT	30	30	17.80%	23.60 ± 2.18	23.60 ± 2.18	0/30	0/30	5	Orbicularis oculi muscle depressor supercilii muscle	At days 6 to 7 postoperatively	0.9% saline	Intraocular canthus	Surgery	1, 3, 6	VSS, VAS
Lee, S.	2018	RCT	15	15	0	34.33 ± 16.99	30.27 ± 10.90	6/9	8/7	25	Forehead area except the supraorbital rim	Within 5 days of primary closure	No treatment	Forehead	Trauma	1, 6	VSS, scar width
Zelken, J.	2016	RCT	26	26	0	53.50 ± 20.40	53.50 ± 20.40	16/10	16/10	40	Frontalis muscle	10 days before surgery	0.9% saline	Forehead	Surgery	6	VAS
Chang, C. S.	2014	RCT	30	28	3.30%	24.70 ± 7.16	21.87 ± 8.00	12/18	14/14	25	Administered to the orbicularis oris muscle 5 mm either side of the wound below the nasal base and above the vermillion border	Immediately after skin closure	0.9% saline	Upper lip	Birth defects	6	VAS, VSS, scar width
Chang, C. S.	2014	RCT	30	29	1.70%	3.13 ± 0.37	3.17 ± 0.25	19/11	19/10	25	Subjacent orbicularis oris muscle	Immediately after skin closure	0.9% saline	Upper lip	Birth defects	6	VAS, VSS, scar width
Ziade, M.	2013	RCT	11	13	20%	38.91 ± 14.52	46.00 ± 24.02	8/3	7/6	10	Facial muscles	Within 72 h postoperatively	No treatment	Face	Trauma	12	VAS, VSS, PSAS, OSAS
Gassner, H. G.	2006	RCT	16	15	26%	62.00 ± 18.20	60.20 ± 16.70	10/6	11/4	75	Musculature adjacent to the wound in a diameter of approximately 1–3 cm around the wound edges	Within 24 h after wound closure	0.9% saline	Forehead	Trauma	6	VAS
Kim, S. H.	2019	RCT	24	21	25%	38.79 ± 13.01	34.67 ± 12.84	11/13	11/10	25	Around the sutured site within a 0.5 cm distance	Within postoperative days 5	0.9% saline	Forehead	Trauma	1, 3, 6	VAS, SBSES, PSAS OSAS

Abbreviations: F, female; M, male; MMSS, Modified Manchester Scar Scale; OSAS, Observer Scar Assessment Scale; PSAS, Patient Scar Assessment Scale; RCT, randomized controlled trials; SBSES, Scar Evaluation Scale; VAS, visual analog scale; VSS, Vancouver Scar Scale.

### 3.2 Quality Evaluation

**FIGURE 2:**
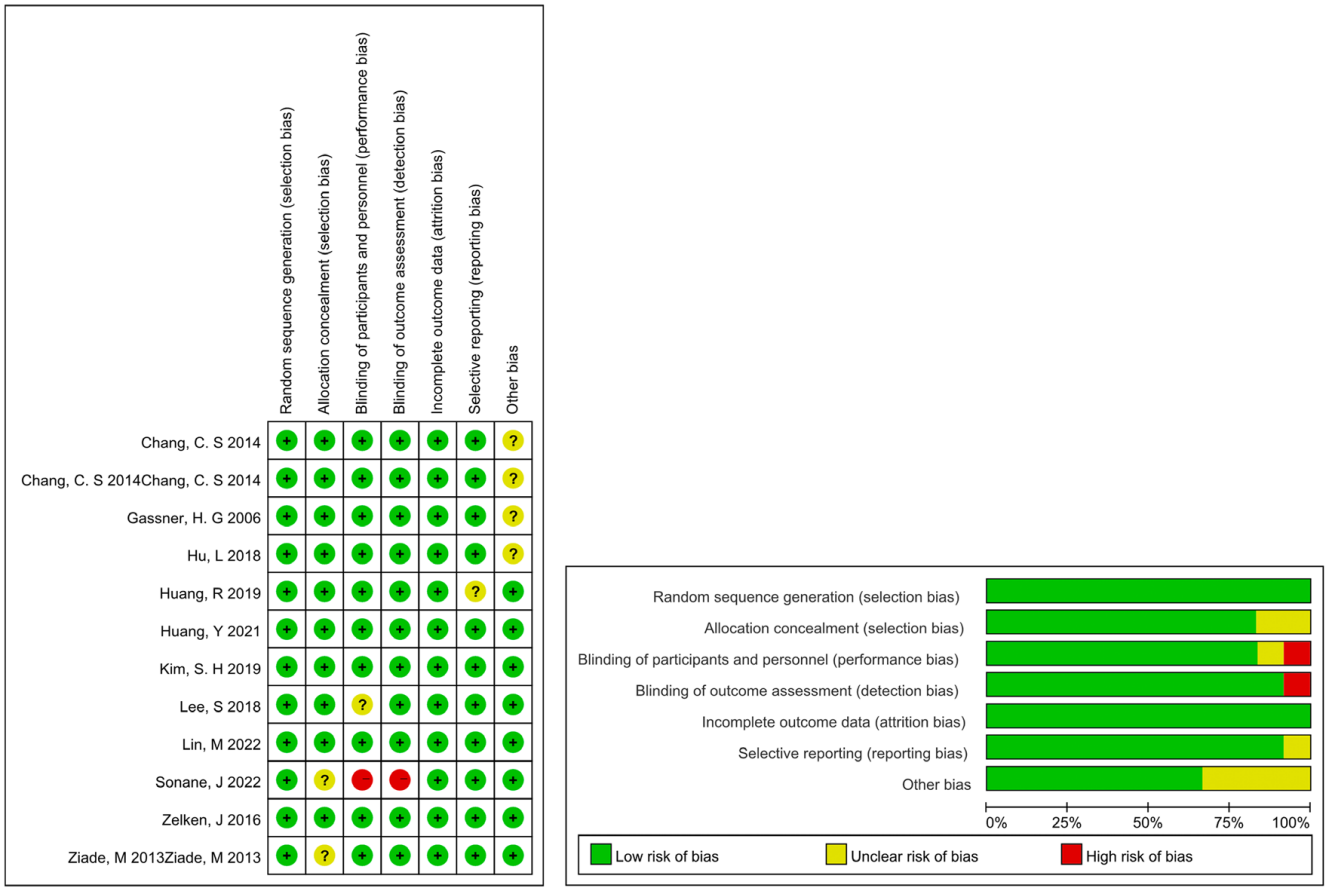
Risk of bias map and risk of bias summary plot. All studies used for analysis assessed risk of bias according to the Cochrane Manual, and the results indicated that the studies were at low risk.

We assessed the risk of bias of all studies used for the analysis according to the Cochrane Manual, and the results indicated that the studies were at low risk. The specific quality evaluation is shown in Table 2 and Figure 2.

**TABLE 2:** Cochrane quality assessment.

Author	Year	Baseline comparability	Random allocation method	Allocation scheme hiding	Blind method	Result data integrity	Selectively report research findings	Other sources of bias
Sonane, J.	2022	✓	Randomized control (random allocation method not described)	Not detailed	Open method	✓	×	×
Hu, L.	2018	✓	Randomized control (random allocation method not described)	✓	Blind method	✓	×	Unspecified
Huang, Y.	2021	✓	Randomized control (random number)	✓	Blind method	✓	×	×
Lin, M.	2022	✓	Randomized control (random allocation method not described)	✓	Blind method	✓	×	×
Huang, R.	2019	✓	Randomized control (random allocation method not described)	✓	Blind method	✓	Unspecified	×
Lee, S.	2018	✓	Randomized control (random allocation method not described)	Not detailed	Blind method	✓	×	×
Zelken, J.	2016	✓	Randomized control (random allocation method not described)	✓	Blind method	✓	×	×
Chang, C. S.	2014	✓	Randomized control (random allocation method not described)	✓	Blind method	✓	×	Unspecified
Chang, C. S.	2014	✓	Randomized control (random allocation method not described)	✓	Blind method	✓	×	Unspecified
Ziade, M.	2013	✓	Randomized control (random allocation method not described)	Not detailed	Blind method	✓	×	×
Gassner, H. G.	2006	✓	Randomized control (random allocation method not described)	✓	Blind method	✓	×	Unspecified
Kim, S. H.	2019	✓	Randomized control (random allocation method not described)	✓	Blind method	✓	×	×

Baseline level			
Variables		*I* ^2^ (%)	*p*
Age WMD (95% CI)	0.06 (−0.13 to 0.24)	0	0.95
Female OR (95% CI)	1.10 (0.73 to 1.67)	0	0.90

Abbreviations: OR, odds ratio; WMD, weighted mean difference.

### 3.3 Outcome

There were statistically significant differences in the scores of visual analog scale (VAS), Vancouver Scar Scale (VSS), scar width, and Observer Scar Assessment Scale (OSAS), and there was no statistically significant difference between Patient Scar Assessment Scale (PSAS) and adverse effects, and there was no publication bias.

#### 3.3.1 Visual Analog Scale

**FIGURE 3:**
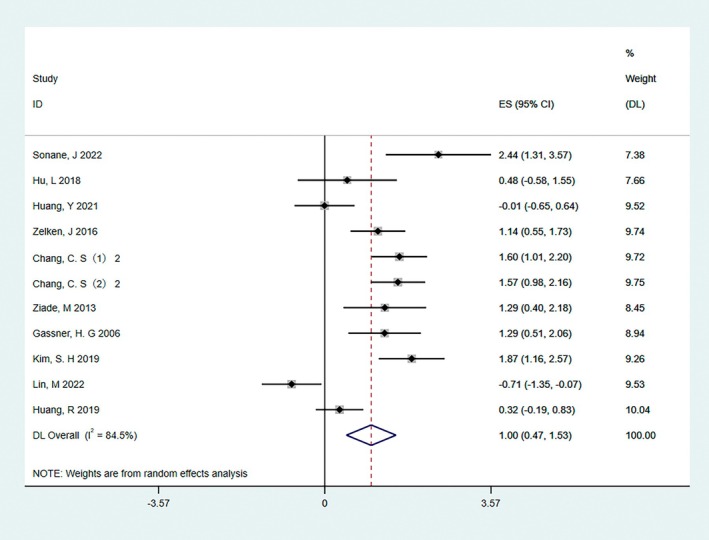
The result of the VAS score. The results showed that the BTA injection group had a higher overall VAS score (SMD = 1.00, 95% CI: 0.47–1.53, *p* = 0.005).

Eleven RCTs with a total of 432 patients used VAS to assess final scar outcomes between the trial and control groups [10, 11, 12, 13, 14, 15, 16, 17, 18, 19, 20]. A preliminary analysis (*I* ^2^ = 84.5%) found some heterogeneity between the included studies; therefore, the random‐effects model was used for statistical analysis. The results showed that the overall VAS score was higher in the BTA injection group than in the control group (SMD = 1.00, 95% CI: 0.47–1.53, *p* = 0.005, Figure 3).

#### 3.3.2 Vancouver Scar Scale

**FIGURE 4:**
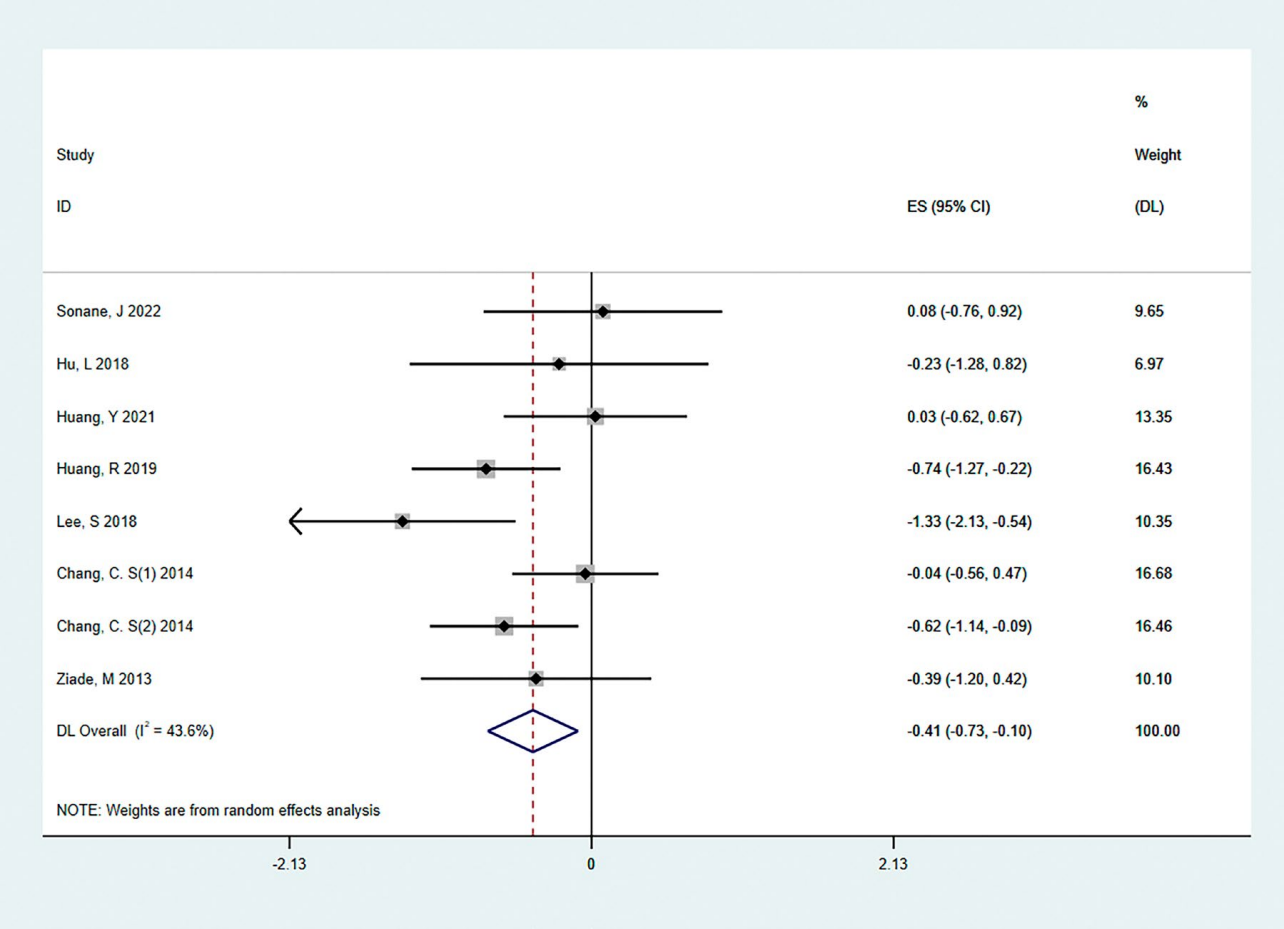
The result of the VSS score. There was some heterogeneity between the studies included in the primary analysis (*I* ^2^ = 43.6%); the results showed that the overall VSS score was lower in the BTA injection group (SMD = −0.41, 95% CI: −0.73 to −0.1, *p* = 0.039).

At present, it is a relatively common scar assessment method in the world, which is measured in four aspects: observation of scar pigmentation, blood circulation, scar texture, and scar thickness without the help of special tools. The inclusion included 304 cases (eight studies) reporting VSS scores, and preliminary analysis suggested some heterogeneity between the included studies (*I* ^2^ = 43.6%); the results showed that the overall VSS score in the BTA group was lower than in the control group (SMD = −0.41, 95% CI: −0.73 to −0.1, *p* = 0.039, Figure 4).

#### 3.3.3 Scar Width

**FIGURE 5:**
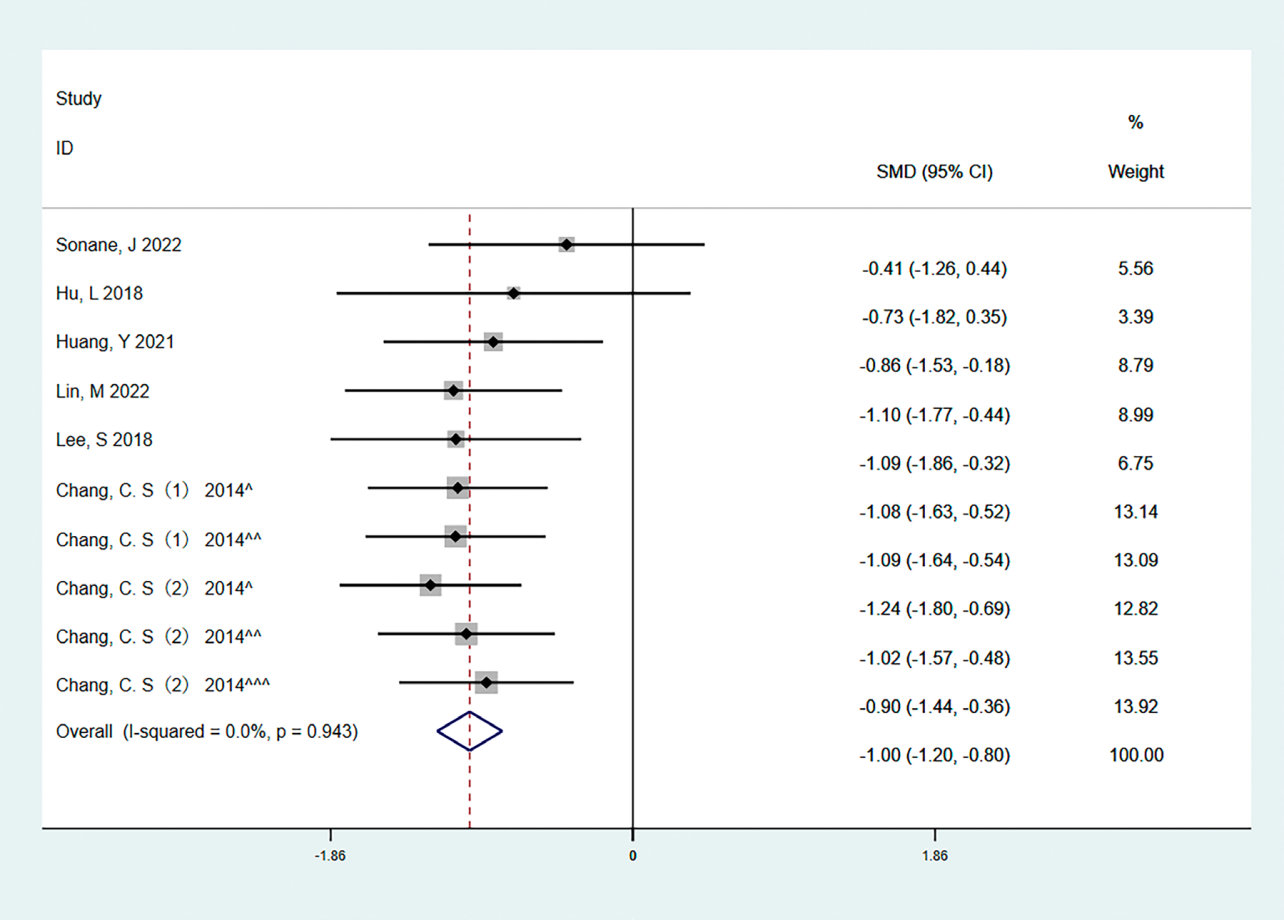
The result of the scar width score. The results of quantitative analysis showed that the facial scar width in the BTA group was lower than that in the control group (SMD = −1.00, 95% CI: −1.20 to −0.80, *p* < 0.05).

The width of the scar can be a visual indication of the degree of scar hyperplasia that has formed after surgery. In seven randomized controlled trials involving 436 cases, scar width was used to assess final scar outcomes between experimental and control groups, and there was no significant heterogeneity in preliminary analysis (*I* ^2^ = 0). The results of quantitative analysis showed that the facial scar width in the BTA group was lower than that in the control group (SMD = −1.00, 95% CI: −1.20 to −0.80, *p* < 0.05, Figure 5).

#### 3.3.4 POSAS

**FIGURE 6:**
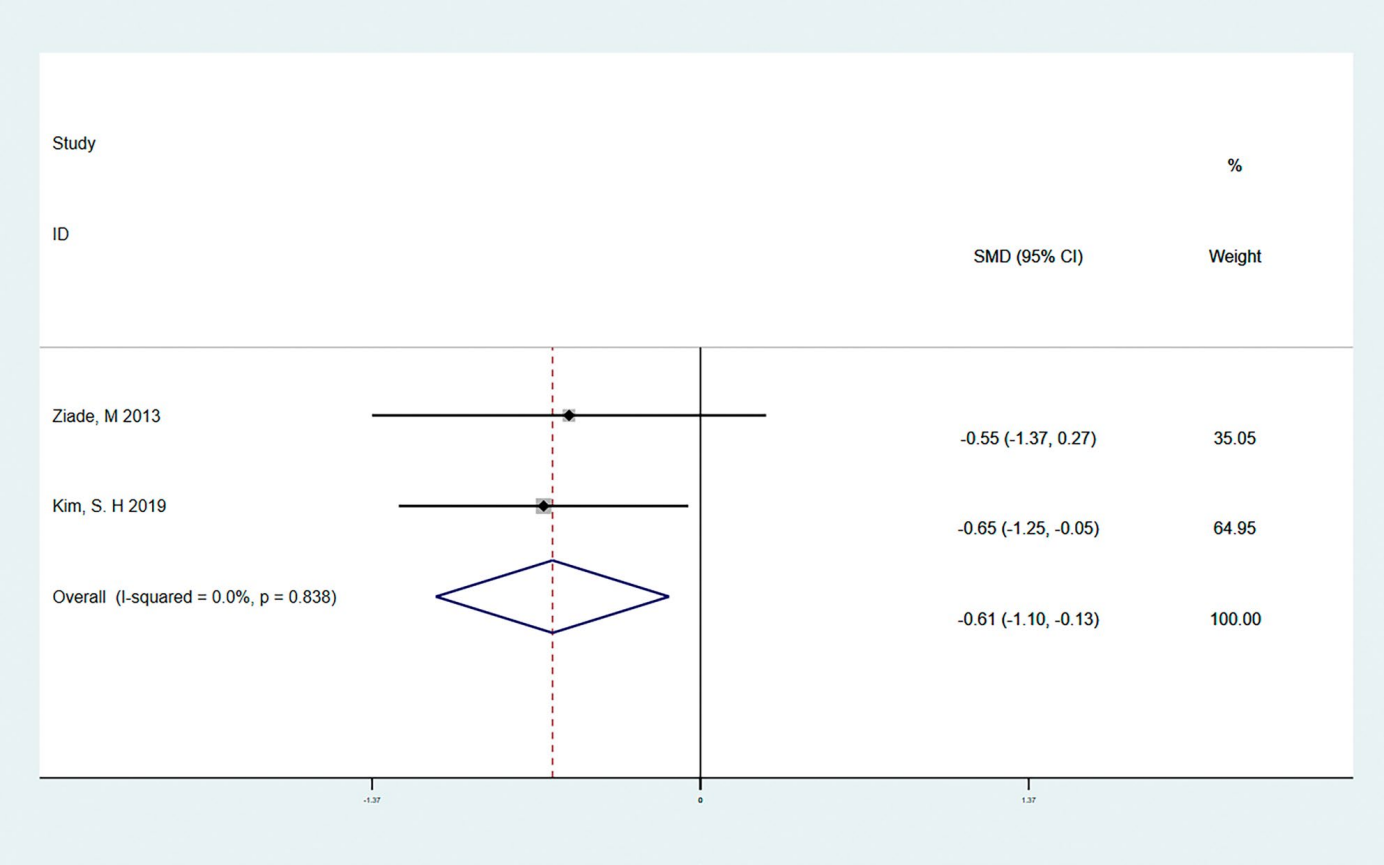
The result of the OSAS score. The results of quantitative analysis showed that the OSAS score of the BTA group was lower than that of the control group, and the difference was statistically significant (SMD = −0.61, 95% CI: −1.0 to −0.13, *p* = 0.013 < 0.05).

**FIGURE 7:**
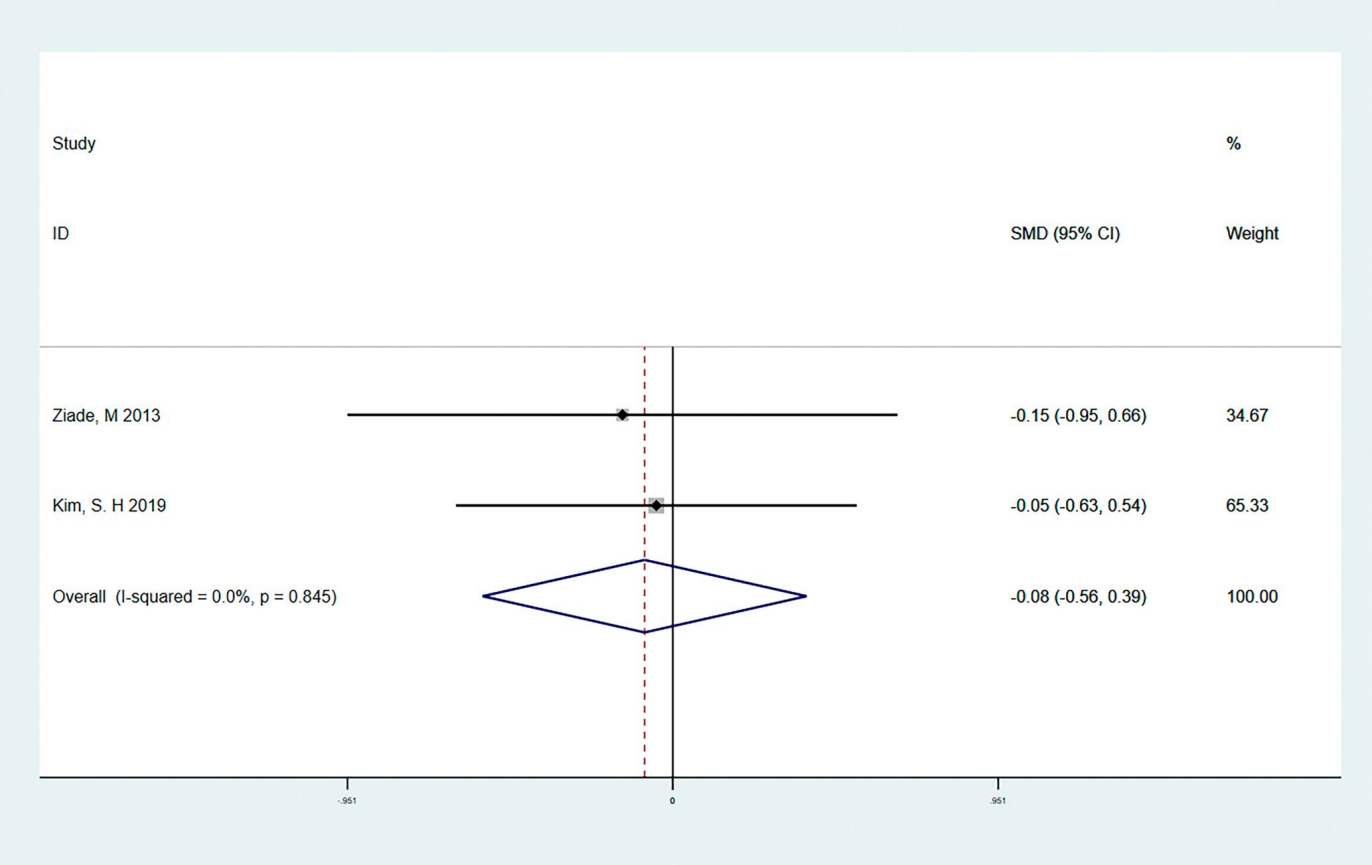
The result of the PSAS score. There was no significant heterogeneity in PSAS (*I* ^2^ = 0.0%, *Q*‐test *p* = 0.845 > 0.1). The results of quantitative analysis showed that there was no significant difference (SMD = −0.08, 95% CI = −0.56 to 0.39, *p* = 0.733 > 0.05).

Among scar assessments, POSAS is currently the most comprehensive and commonly used scar assessment scale, as it is the first scale to integrate the views of the observer and the patient. POSAS consists of two questionnaires, the OSAS and the PSAS, which are used for the observer and the patient, respectively. There was no significant heterogeneity in the preliminary analysis of OSAS (*I* ^2^ = 0.0%, *Q*‐test *p* = 0.838 > 0.1). The results of quantitative analysis showed that the OSAS score of the BTA group was lower than that of the control group, and the difference was statistically significant (SMD = −0.61, 95% CI: −1.0 to −0.13, *p* = 0.013 < 0.05, Figure 6). There was no significant heterogeneity in PSAS (*I* ^2^ = 0.0%, *Q*‐test *p* = 0.845 > 0.1). The results of quantitative analysis showed that there was no significant difference (SMD = −0.08, 95% CI = −0.56 to 0.39, *p* = 0.733 > 0.05, Figure 7).

#### 3.3.5 Adverse Effects

**FIGURE 8:**
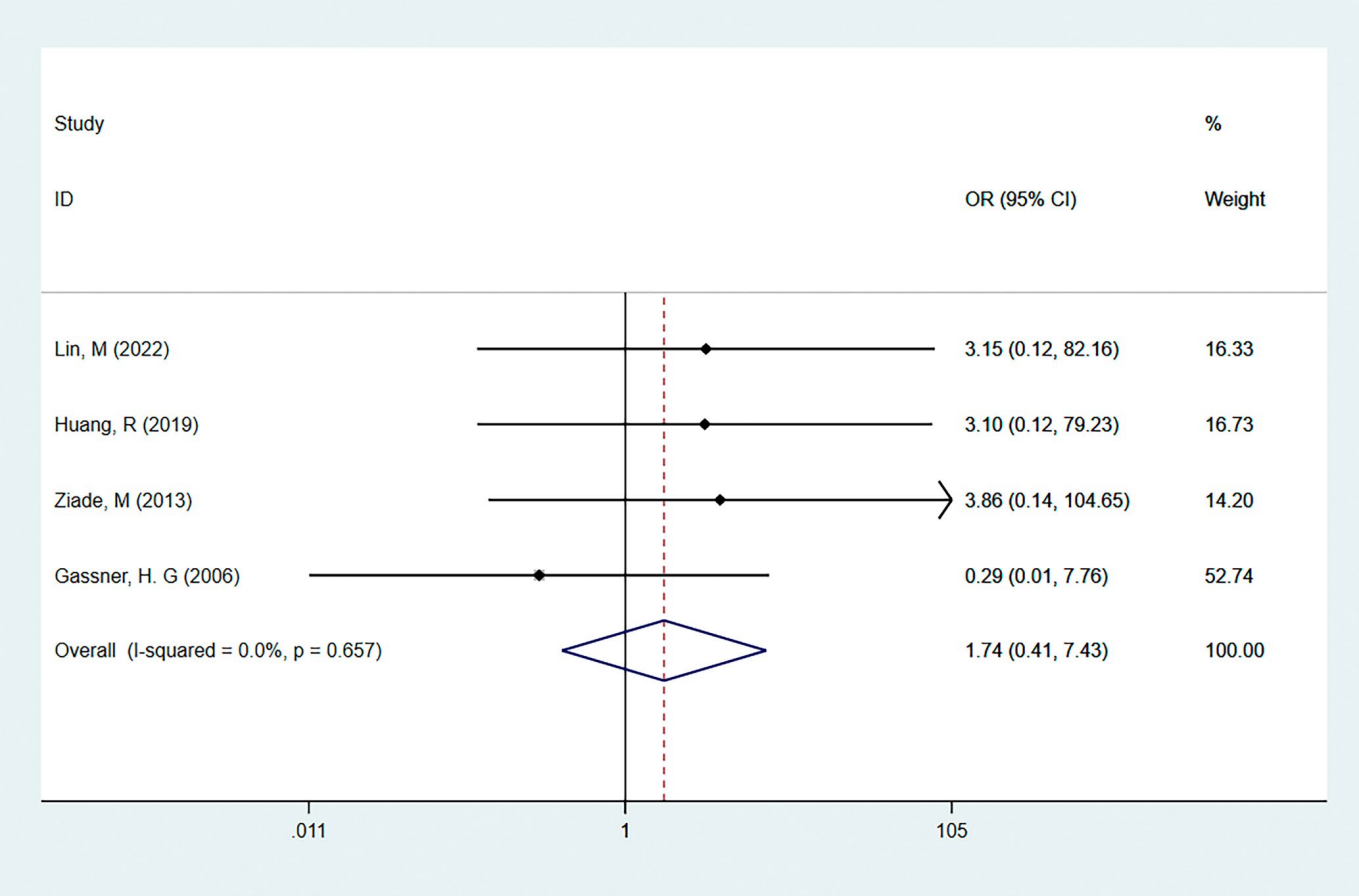
The result of the adverse effects score. There was no significant correlation between BTA injection and the control group (RR = 1.74, 95% CI: 0.41–7.43, *p* = 0.46). Therefore, in summary, BTA injection is safe for postoperative scar management.

Adverse effects were reported in four studies, two of which were ptosis in the BTX group, one was asymmetrical smiling in the BTX group, and one was a mild headache in the control group. Adverse events were analyzed using binary data. After combining the data, a fixed‐effect model was used because there was no significant heterogeneity (*I* ^2^ = 0). There was no significant correlation between BTA injection and the control group (RR = 1.74, 95% CI: 0.41–7.43, *p* = 0.46, Figure 8). Therefore, in summary, BTA injection is safe for postoperative scar management.

### 3.4 Publication Bias

**FIGURE 9:**
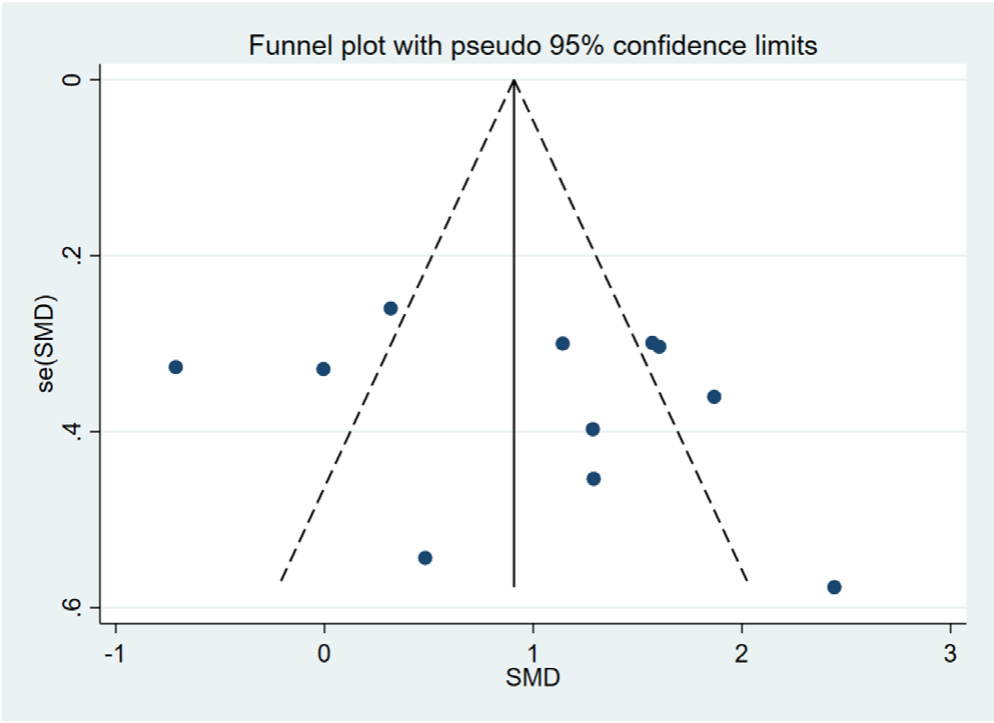
The Begg's test is a test that normalizes the rank correlation between the effect size and the variance of the effect value to determine the severity of publication bias, and the closer it is to zero, the more serious the problem. When VAS, VSS, scar width, and OSAS assessed the effect of BTA, the Begg's test did not find significant publication bias.

Publication bias was assessed using funnel plots and Begg's test. In this meta‐analysis, VAS, VSS, OSAS, and scar width were used as the outcome measure to evaluate the study of BTA inhibiting scar hyperplasia. No significant publication bias was found. The funnel plot was basically symmetrical, and Begg's test showed *p* > 0.05, as shown in Figure 9.

### 3.5 Subgroup Analysis

#### 3.5.1 Outcome Measures

Subgroup analyses were performed for the inclusion of three studies based on differences in saline or no treatment in the control group. The results are shown in Table 3. VSS, VAS, and scar width were all statistically significant; the results were consistent with the previous results.

**TABLE 3:** Subgroup analysis.

Outcome measures	Number of included studies	Heterogeneity test results		Effect model	Meta‐analysis results	*p*
		*I* ^2^	*p*		Effect size (95% CI)	
VAS	12	84.5%	0.00	Random effect	1.00 (0.47 to 1.53)	0.005
BTA vs. Saline	11	72.9%	0.00	Random effect	0.91 (0.55 to 1.27)	0.007
BTA vs. Blank	1	—	—	—	1.29 (0.40 to 2.18)	0.004
VSS	8	43.6%	0.09	Random effect	−0.41 (−0.73 to −0.1)	0.039
BTA vs. Saline	6	26.9%	0.23	Fixed effect	−0.33 (−0.58 to −0.07)	0.011
BTA vs. Blank	2	58.0%	0.12	Fixed effect	−0.893 (−1.46 to −0.32)	0.002
Scar width	10	0%	0.94	Fixed effect	−1.00 (−1.20 to −0.80)	0.000
BTA vs. Saline	9	0%	0.91	Fixed effect	−1.00 (−1.20 to −0.79)	0.000
BTA vs. Blank	1	—	—	—	−1.09 (−1.86 to −0.32)	0.006

Abbreviations: BTX, botulinum toxin; VAS, visual analog scale; VSS, Vancouver Scar Scale.

## 4 Discussion

Wound healing is a complex biological process that is typically divided into three main stages: inflammatory, proliferative, and remodeling. Inflammatory phase: When the skin is damaged, the body immediately initiates an inflammatory response. The main purpose of this stage is to remove damaged tissue, prevent infection, and start the healing process. The inflammatory response is caused by blood vessel dilation and increased vascular permeability, which allows white blood cells and other immune cells in the blood to enter the damaged area. These cells remove bacteria and dead tissue and release pro‐inflammatory mediators that facilitate the healing process. Proliferative stage: After the inflammatory stage, the wound begins to enter the proliferative stage. At this stage, new blood vessels begin to grow (angiogenesis), providing oxygen and nutrients to the damaged area. At the same time, fibroblasts begin to move to the damaged area and produce collagen, the main structural protein of the skin, forming new connective tissue. This process causes the wound to gradually close and form granulation tissue, which is ready to proceed to the next stage. Remodeling stage: After the hyperplastic stage, the wound enters the remodeling stage. At this stage, the newly formed tissue is gradually reshaped and strengthened. The fibroblasts continue to produce collagen and begin to rearrange to strengthen the structure of the wound. This process can take several months or even more than a year until the wound is fully healed and at full strength. These stages are usually sequential and overlapping, rather than strictly separate. The speed and quality of wound healing are influenced by a variety of factors, including the size and depth of the wound, the health status of the individual, nutritional status, age, and environmental factors [22, 23, 24, 25].

Scars, especially on exposed body parts, are difficult to hide and may cause psychological distress. Therefore, it is very important to find an effective and convenient scar prevention and treatment method. This can well improve the psychological condition of patients and also play a positive role in the primary disease [26].

In recent years, many advances have been made in the mechanism of BTA in the prevention and treatment of scars. Prophylactic injection of BTXA effectively reduces scar formation in skin wounds. This is related to the fact that BTA can inhibit the polarization of M1 macrophages, thereby inhibiting the promotion of tissue inflammation and scar formation by M1 macrophages in the early stage of wound healing, which is related to the JAK2/STAT1 and IκB/NFκB pathways [27]. By blocking the activation of JNK, ERK, and p38 MAPK, BTA can inhibit the production of NO and TNF‐α in RAW264.7 macrophages induced by lipopolysaccharide at the transcriptional level and inhibit the expression of macrophages, thus reducing the inflammation in the process of scar production and inhibiting scars [28]. Botulinum toxin is a neuromuscular blocker that causes muscle paralysis and relaxation by blocking the transmission of nerve impulses to the muscles. During wound healing, excessive muscle tension may lead to the formation of scar tissue. Thus, reducing muscle tone by injecting botox, which reduces tension in the area of trauma, may help prevent scar formation and is thought to be the main mechanism for its traditional clinical use [2, 29].

The efficacy and safety of BTA in the treatment of postoperative scars were evaluated by six rating scales. According to the analysis of the 12 included studies, the PSAS scale was not statistically significant, and the number of studies using the MMSS and SBSES scales as outcome indicators was too small to conduct a joint effect size analysis. Other scales were statistically significant. In addition to the scales, scar width was also used to assess the effect of BTA on scars. By analysis, the scar width of the experimental group was smaller than that of the control group, which also demonstrated the effectiveness of BTA in postoperative scar management. Adverse events were analyzed according to binary data to evaluate the safety of BTA, and the results showed that there was no statistically significant difference between the experimental group and the control group. No serious complications occurred, and minor complications disappeared spontaneously during the follow‐up. In conclusion, BTA was safe. In addition, the control group was divided into normal saline and no treatment for subgroup analysis, and finally obtained the same results.

### 4.1 Strengthen and Limitations

More RCTs were included, the sample size was expanded, the stability and reliability of the results were improved, and subgroup analyses were performed according to the differences between the control groups to explore the differences in effects between different subgroups. The reason for the different doses of BTX in each group was not specifically analyzed for heterogeneity.

## 5 Conclusions

Botulinum toxin is effective and safe in postoperative scar management. The results of the meta‐analysis showed that botulinum toxin could effectively reduce the symptoms of redness, swelling, sclerosis, and pain of postoperative scars and promote the healing and smoothing of scars. In addition, the use of botulinum toxin in postoperative scar management has no significant serious side effects or safety concerns. Therefore, botox can be used as an effective and safe method for postoperative scar management. However, more clinical studies are needed to further validate its efficacy and safety.

## Conflicts of Interest

Publicly available datasets from PubMed, Embase, MEDLINE, the Cochrane Library (CENTRAL), and Web of Science were analyzed in this study. Detailed information on the original studies is provided in the Supporting Information. For further inquiries, please contact the corresponding author.

## Supporting information


Data S1

Data S2

## Data Availability

Publicly available datasets were analyzed in this study. The data were sourced from PubMed, Embase, MEDLINE, the Cochrane Library (CENTRAL), and Web of Science. Detailed information on the original studies can be found in the Supporting Information. For further inquiries, contact the corresponding author. Additional details are available in the Supporting Information.

## References

[1] S. H. Lee , H. J. Min , Y. W. Kim , and Y. W. Cheon , “The Efficacy and Safety of Early Postoperative Botulinum Toxin A Injection for Facial Scars,” Aesthetic Plastic Surgery 42, no. 2 (2018): 530–537. 29214336 10.1007/s00266-017-1008-7

[2] H. G. Gassner and D. A. Sherris , “Chemoimmobilization: Improving Predictability in the Treatment of Facial Scars,” Plastic and Reconstructive Surgery 112 (2003): 1464–1466. 14504533 10.1097/01.PRS.0000081073.94689.DB

[3] H. S. Jeong , B. H. Lee , H. M. Sung , et al., “Effect of Botulinum Toxin Type A on Differentiation of Fibroblasts Derived From Scar Tissue,” Plastic and Reconstructive Surgery 136, no. 2 (2015): 171e–178e. 10.1097/PRS.0000000000001438 26218391

[4] D. A. Sherris and H. G. Gassner , “Botulinum Toxin to Minimize Facial Scarring,” Facial Plastic Surgery 18, no. 1 (2002): 35–39. 11823931 10.1055/s-2002-19825

[5] P. J. Carniol , L. Meshkov , and L. D. Grunebaum , “Laser Treatment of Facial Scars,” Current Opinion in Otolaryngology & Head and Neck Surgery 19 (2011): 283–288. 21659876 10.1097/MOO.0b013e32834896b9

[6] D. H. Jung , G. S. Medikeri , G. U. Chang , and S. M. Hyun , “Surgical Techniques for the Correction of Postrhinoplasty Depressed Scars on the Nasal Tip,” JAMA Facial Plastic Surgery 17 (2015): 405–412. 26379006 10.1001/jamafacial.2015.0911

[7] J. Gallagher , I. W. Goldfarb , H. Slater , and M. Rogosky‐Grassi , “Survey of Treatment Modalities for the Prevention of Hypertrophic Facial Scars,” Journal of Burn Care & Rehabilitation 11 (1990): 118–120. 2335548 10.1097/00004630-199003000-00005

[8] B. Chowdhury , M. Kassir , J. Salas‐Alanis , et al., “Laser in Surgical Scar Clearance: An Update Review,” Journal of Cosmetic Dermatology 20 (2021): 3808–3811, 10.1111/jocd.14325. 34213802

[9] P. Sun , X. Lu , H. Zhang , and Z. Hu , “The Efficacy of Drug Injection in the Treatment of Pathological Scar: A Network Meta‐Analysis,” Aesthetic Plastic Surgery 45, no. 2 (2021): 791–805. 31853608 10.1007/s00266-019-01570-8

[10] L. Hu , Y. Zou , S. J. Chang , et al., “Effects of Botulinum Toxin on Improving Facial Surgical Scars: A Prospective, Split‐Scar, Double‐Blind, Randomized Controlled Trial,” Plastic and Reconstructive Surgery 141, no. 3 (2018): 646–650. 29481395 10.1097/PRS.0000000000004110

[11] J. Sonane , R. K. Sharma , J. R. John , and R. Sharma , “Botulinum Toxin for a Better Scar in Cleft Lip Surgery: A Prospective Randomized Control Trial,” Journal of Craniofacial Surgery 33, no. 1 (2022): 198–202. 34267122 10.1097/SCS.0000000000007836

[12] Y. L. Huang , C. G. Wallace , Y. C. Hsiao , et al., “Botulinum Toxin to Improve Lower Blepharoplasty Scar: A Double‐Blinded, Randomized, Vehicle‐Controlled Clinical Trial,” Aesthetic Surgery Journal 41, no. 9 (2021): 1003–1010, 10.1093/asj/sjab024. 34128526

[13] M. J. Lin , D. M. Bernstein , R. L. Torbeck , D. P. Dubin , J. D. Rosenberg , and H. Khorasani , “Botulinum Toxin Improves Forehead Scars After Mohs Surgery: A Randomized, Double‐Blinded, Controlled Study,” Journal of the American Academy of Dermatology 86, no. 4 (2022): 964–966. 33848604 10.1016/j.jaad.2021.03.110

[14] R. L. Huang , C. K. Ho , M. Tremp , Y. Xie , Q. Li , and T. Zan , “Early Postoperative Application of Botulinum Toxin Type A Prevents Hypertrophic Scarring After Epicanthoplasty: A Split‐Face, Double‐Blind, Randomized Trial,” Plastic and Reconstructive Surgery 144, no. 4 (2019): 835–844, 10.1097/PRS.0000000000006069. 31568286

[15] J. Zelken , S. Y. Yang , C. S. Chang , et al., “Donor Site Aesthetic Enhancement With Preoperative Botulinum Toxin in Forehead Flap Nasal Reconstruction,” Annals of Plastic Surgery 77, no. 5 (2016): 535–538. 26418784 10.1097/SAP.0000000000000625

[16] C. S. Chang , C. G. Wallace , Y. C. Hsiao , C. J. Chang , and P. K. T. Chen , “Botulinum Toxin to Improve Results in Cleft Lip Repair: A Double‐Blinded, Randomized, Vehicle‐Controlled Clinical Trial,” PLoS One 9, no. 12 (2014): e115690. 25541942 10.1371/journal.pone.0115690 PMC4277415

[17] C. S. Chang , C. G. Wallace , Y. C. Hsiao , C. J. Chang , and P. K. T. Chen , “Botulinum Toxin to Improve Results in Cleft Lip Repair,” Plastic and Reconstructive Surgery 134, no. 3 (2014): 511–516. 25158709 10.1097/PRS.0000000000000416

[18] M. Ziade , S. Domergue , D. Batifol , et al., “Use of Botulinum Toxin Type A to Improve Treatment of Facial Wounds: A Prospective Randomised Study,” Journal of Plastic, Reconstructive & Aesthetic Surgery 66, no. 2 (2013): 209–214. 10.1016/j.bjps.2012.09.012 23102873

[19] H. G. Gassner , A. E. Brissett , C. C. Otley , et al., “Botulinum Toxin to Improve Facial Wound Healing: A Prospective, Blinded, Placebo‐Controlled Study,” Mayo Clinic Proceedings 81, no. 8 (2006): 1023–1028. 16901024 10.4065/81.8.1023

[20] S. H. Kim , S. J. Lee , J. W. Lee , H. S. Jeong , and I. S. Suh , “Clinical Trial to Evaluate the Efficacy of Botulinum Toxin Type A Injection for Reducing Scars in Patients With Forehead Laceration: A Double‐Blinded, Randomized Controlled Study,” Medicine (Baltimore) 98, no. 34 (2019): e16952. 31441893 10.1097/MD.0000000000016952 PMC6716761

[21] D. Moher , L. Shamseer , M. Clarke , et al., “Preferred Reporting Items for Systematic Review and Meta‐Analysis Protocols (PRISMA‐P) 2015 Statement,” Systematic Reviews 4, no. 1 (2015): 1, 10.1186/2046-4053-4-1. 25554246 PMC4320440

[22] D. A. Sherris , W. F. Larrabee, Jr. , and C. S. Murakami , “Management of Scar Contractures, Hypertrophic Scars, and Keloids,” Otolaryngologic Clinics of North America 28, no. 5 (1995): 1057–1068. 8559572

[23] J. L. Burns , J. S. Mancoll , and L. G. Phillips , “Impairments to Wound Healing,” Clinics in Plastic Surgery 30, no. 1 (2003): 47–56. 12636215 10.1016/s0094-1298(02)00074-3

[24] O. A. Peña and P. Martin , “Cellular and Molecular Mechanisms of Skin Wound Healing,” Nature Reviews Molecular Cell Biology 25 (2024): 599–616. 38528155 10.1038/s41580-024-00715-1

[25] S. G. Hameedi , A. Saulsbery , and O. O. Olutoye , “The Pathophysiology and Management of Pathologic Scarring—A Contemporary Review,” Advances in Wound Care 14 (2025): 48–64. 38545753 10.1089/wound.2023.0185 PMC11839539

[26] B. M. Alessandri , J. A. Arellano , A. Scarabosio , et al., “The Effect of Fat Grafting on Scars Hyperpigmentation: A Systematic Review and Meta‐Analysis,” Aesthetic Plastic Surgery 48, no. 5 (2024): 989–998. 38286897 10.1007/s00266-023-03828-8

[27] Y. P. Zou , X. F. Shan , J. X. Qiu , L. N. Wang , R. L. Xiang , and Z. G. Cai , “Botulinum Toxin Type A Inhibits M1 Macrophage Polarization by Deactivation of JAK2/STAT1 and IκB/NFκB Pathway and Contributes to Scar Alleviation in Aseptic Skin Wound Healing,” Biomedicine & Pharmacotherapy 174 (2024): 116468. 38518603 10.1016/j.biopha.2024.116468

[28] Y. J. Kim , J. H. Kim , K. J. Lee , et al., “Botulinum Neurotoxin Type A Induces TLR2‐Mediated Inflammatory Responses in Macrophages,” PLoS One 10, no. 4 (2015): e0120840. 25853816 10.1371/journal.pone.0120840 PMC4390353

[29] J. Y. Chan , L. H. Liu , and W. I. Wei , “Early Prediction of Anastomotic Leakage After Free Jejunal Flap Reconstruction of Circumferential Pharyngeal Defects,” Journal of Plastic, Reconstructive & Aesthetic Surgery 66, no. 3 (2013): 376–381. 10.1016/j.bjps.2012.09.035 23102872

