# Supplementary material for: A Meta‐Analysis of the Efficacy and Safety of Botulinum Toxin Type A for the Management of Scars After Facial Surgery
Source: J Cosmet Dermatol. 2025 Mar 17;24(3):e70111. doi: 10.1111/jocd.70111 (PMC11914370; doi:10.1111/jocd.70111)
Supplement: Supplementary file 1 — Data S1 [file JOCD-24-e70111-s001.docx]

Sample size of each study included in the meta-analysis :.

| Year | Design | Total |  | Loss rate | Age（mean±SD) | | Sex（M/F） | | Concentration | Injection Site | Injection time | Control | Wound | The type of wound | Follow-up | Outcome indicators： |
| --- | --- | --- | --- | --- | --- | --- | --- | --- | --- | --- | --- | --- | --- | --- | --- | --- |
|  |  | Experience | Control |  | Experience | Control | Experience | Control |  |  |  |  |  |  |  |  |
| 2022 | RCT | 12 | 10 | 21% | 6.91 ± 0.65 | 7 ± 0.38 | （6/6） | （6/4） | 1 | adjacent orbicularis oris muscle | immediately after completion of cleft lip repair. | 0.9% saline | Upper lip | Birth defects | 6 | VAS, VSS，Scar width |
| 2018 | RCT | 7 | 7 | 12.50% | 12.29±11.64 | 12.29±11.64 | （7/7） | （7/7） | 33.7 | 5 mm on either side of the wound | Immediately after wound closure | 0.9% saline | face | Surgery | 6 | VAS, VSS，Scar width |
| 2021 | RCT | 18 | 19 | 7.50% | 61.56 ± 8.14 | 61.56 ± 8.14 | （2/16） | （4/15） | 7.5 | lateral orbicularis oculi muscles | immediately after completion of surgery | 0.9% saline | Lower eyelids | Surgery | 7.10 ± 8.03：6.59 ± 8.36 | VAS, VSS，Scar width |
| 2022 | RCT | 20 | 20 | 0 | 58.08 ±21.54 | 59 .00±25.53 | （12/8） | （11/9） | 50 | forehead | immediately following the closure of Mohs defects | 0.9% saline | forehead | Surgery | 1weak，3weaks，6month | MMSS, VAS, Scar width |
| 2019 | RCT | 30 | 30 | 17.80% | 23.60±2.18 | 23.60±2.18 | （0/30） | （0/30） | 5 | Orbicularis oculi muscle depressor supercilii muscle | at days 6 to 7 postoperatively | 0.9% saline | Intraocular canthus | Surgery | 1，3，6 | VSS, VAS |
| 2018 | RCT | 15 | 15 | 0 | 34.33±16.99 | 30.27±10.90 | （6/9） | (8/7） | 25 | Forehead area except the supraorbital rim | within 5 days of primary closure | no treatment | forehead | Trauma | 1，6 | VSS ，Scar width |
| 2016 | RCT | 26 | 26 | 0 | 53.50±20.40 | 53.50±20.40 | （16/10） | （16/10） | 40 | frontalis muscle | 10 days before surgery | 0.9% saline | Forehead | Surgery | 6 | VAS |
| 2014 | RCT | 30 | 28 | 3.30% | 24.70±7.16 | 21.87±8.00 | （12/18） | (14/14） | 25 | administered to the orbicularis oris muscle 5 mm either side of the wound below the nasal base and above the vermillion border | Immediately after skin closure | 0.9% saline | Upper lip | Birth defects | 6 | VAS, VSS, Scar width |
| 2014 | RCT | 30 | 29 | 1.70% | 3.13±0.37 | 3.17±0.25 | （19/11） | （19/10） | 25 | Subjacent orbicularis oris muscle | Immediately after skin closure | 0.9% saline | Upper lip | Birth defects | 6 | VAS, VSS, Scar width |
| 2013 | RCT | 11 | 13 | 20% | 38.91±14.52 | 46.00±24.02 | （8/3） | （7/6） | 10 | facial muscles | within 72h postoperatively | no treatment | Face | Trauma | 12 | VAS, VSS, PSAS, OSAS |
| 2006 | RCT | 16 | 15 | 26% | 62.00±18.20 | 60.20±16.70 | （10/6） | （11/4） | 75 | musculature adjacent to the wound in a diameter of approximately 1 to 3 cm around the wound edges | within 24 hours after wound closure | 0.9% saline | Forehead | Trauma | 6 | VAS |
| 2019 | RCT | 24 | 21 | 25% | 38.79±13.01 | 34.67±12.84 | （11/13） | （11/10) | 25 | around the sutured site within a 0.5 cm distance | Within postoperative days 5 | 0.9% saline | forehead | Trauma | 1，3，6 | VAS，SBSES, PSAS OSAS |
